# Spread Spectrum Modulation with Grassmannian Constellations for Mobile Multiple Access Underwater Acoustic Channels

**DOI:** 10.3390/s22218518

**Published:** 2022-11-05

**Authors:** Christophe Bernard, Pierre-Jean Bouvet, Beatrice Tomasi

**Affiliations:** 1 L@bISEN Yncréa Ouest, CS 42807, CEDEX 2, 29228 Brest, France; 2 NORCE Norwegian Research Centre, 5008 Bergen, Norway

**Keywords:** underwater communications, multiple access, spread spectrum communication, Grassmannian modulation

## Abstract

The objective of this study is to evaluate Grassmannian constellations combined with a spread spectrum multiple access scheme for underwater acoustic mobile multiple access communication systems. These communication systems enable the coordination of a fleet of Autonomous Underwater Vehicles (AUVs) from a surface or bottom control unit, e.g., a boat. Due to its robustness against phase rotation, the demodulator of Grassmannian constellations uses non-coherent detection, and the main advantage of such modulation lies in the spectrum efficiency gain with respect to conventional differential modulation. The communication system under study in this paper consists of (i), at the transmitter side, a Grassmannian modulation used in an orthogonal spread spectrum multiple access scheme called Multiuser Hyperbolic Frequency Modulation (MU-HFM) and (ii), at the receiver side, a non-coherent array decoder. The modulation and demodulation are presented as well as the considered spreading sequences. Finally, performances of the proposed transmission scheme are evaluated over replayed underwater acoustic channel responses collected at sea by a multi-sensor acoustic acquisition system.

## 1. Introduction

Digital communication systems for Underwater Acoustic (UWA) channels are traditionally affected by long multipath channels (e.g., caused by the reverberation of the acoustic wave on the bottom or the surface of the water), a large Doppler spread, and movement-induced Doppler shifts. The speed of sound in water (*c* = 1500 m·s^−1^) is five orders of magnitude lower than the speed of light in air, and this causes long propagation delays and multipath channels. The communication system can be considered as ultra-wideband, since the center frequency is comparable with the available bandwidth (as an example, in this paper, we consider a center frequency of 27 kHz with a bandwidth equal to 4 kHz). Sound attenuation in water is frequency dependent and time-varying, and together with background acoustic noise, they limit the achievable data rate considerably as indicated in [1,2]. Because of these drawbacks, realizing a communication system that makes a fleet of Autonomous Underwater Vehicles (AUVs) a mobile connected network is a challenging task. An example of the considered scenario consists of multiple AUVs that communicate with a receiver array deployed over a surface control unit, which could be a boat or a buoy. In the following, we consider the access channel between a fleet of $N_{u}$ AUVs transmitting data and a receiver equipped with $N_{r}$ hydrophones situated at the sea surface.

An extensive multipath effect causes inter-symbol interference (ISI) in the received signal that can be addressed either by non-coherent modulation such as Frequency Shift Keying (FSK) [2] or coherent modulation associated with an advanced equalization scheme, like the Decision Feedback Equalizer (DFE) as proposed in [3,4,5]. Another approach to deal with Inter Symbol Interference (ISI) is the use of spread spectrum communication combined with Differential Phase Shift Keying (DPSK) modulation at the price of data rate reduction [6]. This loss in the data rate is accentuated by the insertion of a preamble sequence often required to estimate time-varying Doppler shifts or to detect the start of a data frame. In [7,8], a preamble called a Dual-HFM signal, which consists of up-chirp Hyperbolically Frequency Modulation (HFM) and down-chirp HFM, is used for Doppler shift estimation. In [7], Doppler shift estimation is done by matched filtering at the receiver. This makes it possible to find the positions of the 2 HFM signals. A method based on speed spectrum scanning, which consists of starting from a set of candidate speeds, is used in [8] in order to improve the accuracy of the Doppler shift estimation. Several variants exist on the combination of the preamble, as, for example, in [9], where the preamble is composed of chirp Linear Frequency Modulation (LFM) and chirp HFM. However, the methods cited above do not apply in the case of a multi-user scenario. In the rest of the paper, we will use the method given in [10], which consists in using several filter banks to find the Doppler shift estimation.

To be able to detect and correctly decode multiple users at the receiver, traditional orthogonal multiple access schemes are Time-Division Multiple Access (TDMA), where a time slot is assigned to each user with a guard period, or Code-Division Multiple Access (CDMA), where a specific spreading code is assigned to each user [11]. As an alternative to CDMA, we introduced in [12] a novel multiple access scheme, called MultiUser Hyperbolically Frequency Modulation (MU-HFM), which is based on a set of mutually orthogonal chirp-based waveforms that are robust with respect to Doppler and multipath effects. The objective of this proposed scheme is, on the one hand, to benefit from the robustness of chirps against UWA channel impairments and, on the other hand, to use orthogonality to separate multiple users at the receiver side, using a simple matched filter. However, the MU-HFM scheme proposed in [12] employs DPSK modulation that exhibits low spectral efficiency. In order to increase the data rate of the communication system, we propose in this paper to consider non-coherent Grassmannian modulation whose codeword is generated by the Cube-Split quantizer that was originally introduced in [13,14]. Several other Grassmannian modulations exist in the literature, as in [15,16]. However, this type of modulation requires that the modulation symbols at the receiver are stored. In this paper, we will consider a variant of this modulation scheme that has a lower demodulation complexity at the receiver. In the following, we compare the performance obtained with the Cube-Split modulation with that obtained by a differential phase shift keying modulation that has the same spectral efficiency. We then increase the order of the Grassmannian modulation in order to achieve a higher spectral efficiency, and therefore a larger amount of bits per symbol, to then compensate for the low spectral content typical of spread spectrum communication schemes. Higher order Grassmannian modulations require higher robustness against inter-symbol interference. This is achieved by exploiting the channel spatial diversity processing gain provided by the hydrophone array at the receiver. To the best of the authors’ knowledge, there is no previously published work presenting Grassmannian modulations applied to UWA communication systems, so the main contribution of the paper lies in the use of Grassmannian modulations as an alternative of differential modulation for non-coherent UWA communication in the context of an underwater multiple-user mobile network.

The paper is organized as follows: in Section 2, we introduce the system model and detail the MU-HFM multiple access scheme. The construction of the Grassmannian modulation based on the Cube-Split quantizer and the associated decoding process is summarized in Section 3. In Section 4, we provide the performance obtained by using the underWaterAcousTic channEl Replay benchMARK (Watermark) channel simulator [17], where channel impulse responses collected during shallow water experiments conducted in the roadstead of Brest, France, are used. Finally, conclusions are drawn in Section 5.

This article is an extension of the conference paper [12] with the following additional features:

- A study of a new Grassmanian modulation [13,14] combined with a spread spectrum communication in an UWA channel and a comparison with differential modulations of the DPSK type.
- The implementation of Doppler shift estimation and frame synchronization processes at the receiver side for more realistic communication and a comparison of the MU-HFM spreading sequence against Pseudo-Noise (PN) sequence as preamble for Doppler shift estimation.

## 2. System Model

### 2.1. Mathematical Notations

In the following, *j* denotes the unit imaginary number, ${\left||\cdot |\right|}_{2}$ the Euclidean norm, 〈·〉 the scalar product, ${\left(\cdot \right)}^{*}$ the complex conjugate, ${\left(\cdot \right)}^{T}$ the transpose, and u∗v the convolution product between *u* and *v*.

### 2.2. Transmitter

We assume a multi-user spread-spectrum transmission scheme with a baseband transmit signal per user $i\in [1,N_{u}]$ expressed by

(1) $$s_{i}\left(t\right)=\sum_{k=1}^{N_{s}}d_{i,k}g_{i}\left(t-kT_{s}\right),$$

with $N_{s}$ as the number of symbols, and $d_{i,k}$ as the *k*-th modulated symbol. Each modulated symbol is assumed to be a preliminary bit interleaved and encoded with a Forward Error Correction (FEC) code of rate $R_{C}$ in order to increase robustness against channel impairments. For user *i*, $T_{s}$ is the waveform duration, which, in the case of a spread spectrum scheme, also corresponds to the symbol duration, and $g_{i}\left(t\right)$ is the spreading signal.

In order to provide robustness against the distortions within the UWA channel, the spreading signals are constructed from the MU-HFM scheme, originally presented in [12], that consists of HFM-based signals combined in a way to ensure mutually orthogonality. The starting point of the construction is a family of narrow-band HFM signals noted by ${\left\{c_{i}\left(t\right)\right\}}_{i=1}^{N_{u}}$ and defined as follows:

(2) $$c_{i}\left(t\right)=\frac{1}{\sqrt{T_{s}}}e^{-j2\pi k\log\left(1-\frac{t}{it_{0}}\right)\zeta i^{2}},$$

where $t_{0}=\frac{T_{s}\left(f_{h}+f_{l}\right)}{2(f_{h}-f_{l})}$, $k=\frac{T_{s}f_{l}f_{h}}{f_{h}-f_{l}}$ is the signal slope, $f_{l}$ and $f_{h}$ are, respectively, the start and stop frequencies, where *B* denotes the signal bandwidth, and $T_{s}$ the waveform duration. The parameter ζ∈R influences the bandwidth of the different $c_{i}\left(t\right)$ and is determined by using Simpson’s method [18] in order to ensure mutual orthogonality between $c_{i}\left(t\right)$ values [12].

Finally, the different $c_{i}\left(t\right)$ values are combined with an HFM signal over the entire bandwidth. This makes it possible to have a larger $BT_{s}$ time-frequency product and to have a better correlation peak for synchronization [9]. To keep the mutual orthogonality across $c_{i}\left(t\right)$, we use the Gram-Schmidt process [19] given by

(3) $$g_{i}\left(t\right)=e_{i}\left(t\right)=c_{i}\left(t\right)+\alpha_{i}e_{i-1}\left(t\right),$$

where

(4) $$\alpha_{i}=-\frac{\langle c_{i}\left(t\right),e_{i-1}\left(t\right)\rangle }{\left|\right|e_{i-1}\left(t\right){\left|\right|}_{2}^{2}}=-\frac{\int_{\frac{-T_{s}}{2}}^{\frac{T_{s}}{2}}c_{i}\left(t\right)e_{i-1}^{*}\left(t\right)dt}{\left|\right|e_{i-1}\left(t\right){\left|\right|}_{2}^{2}},$$

and $e_{0}\left(t\right)$ is a full bandwidth HFM signal defined as

(5) $$e_{0}\left(t\right)=\left\{\begin{array}{cc} \cos(-2\pi \left(k\log\left(1-\frac{t}{t_{0}}\right)+\frac{f_{l}+f_{h}}{2}t\right)) & \mathrm{if}\;\frac{-T_{s}}{2}\leq t\leq \frac{T_{s}}{2}\\ 0 & \mathrm{otherwise}. \end{array}\right.$$

**Figure 1:**
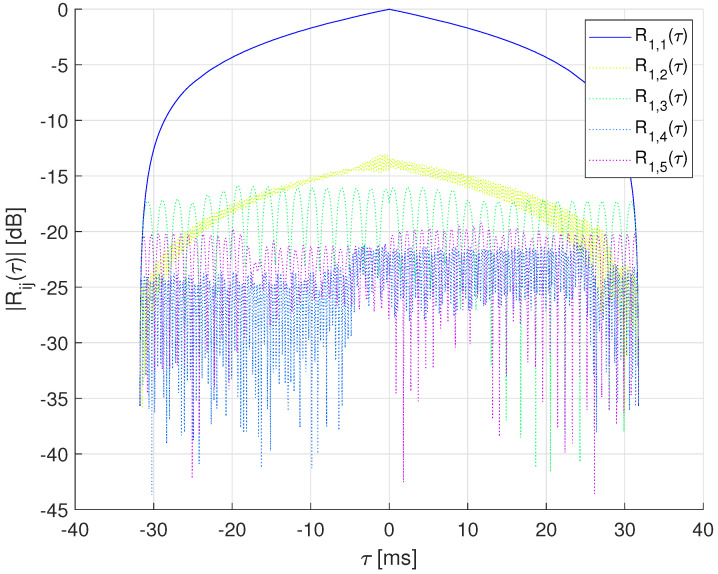
Cross-correlation functions [20] for $g_{1}\left(t\right)$ with $T_{s}=31.75$ ms.

**Figure 2:**
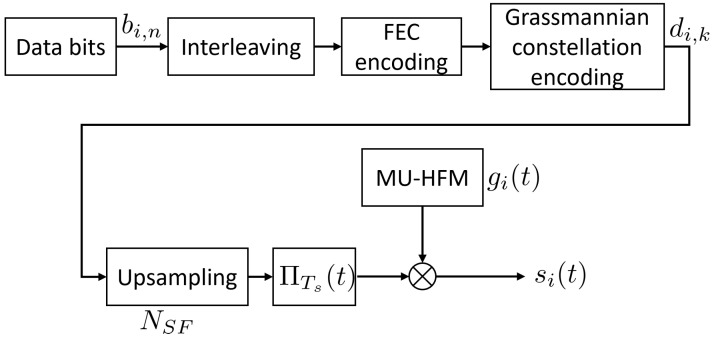
Transmitter diagram, where $N_{SF}$ denotes the spreading factor, and $\Pi_{T_{s}}\left(t\right)$ is the rectangular function of duration $T_{s}$.

Figure 1 shows an example of auto and cross-correlation functions for the signals $g_{i}\left(t\right)$, whereas Figure 2 provides a diagram of the transmitter processing.

### 2.3. Receiver Architecture

**Figure 3:**
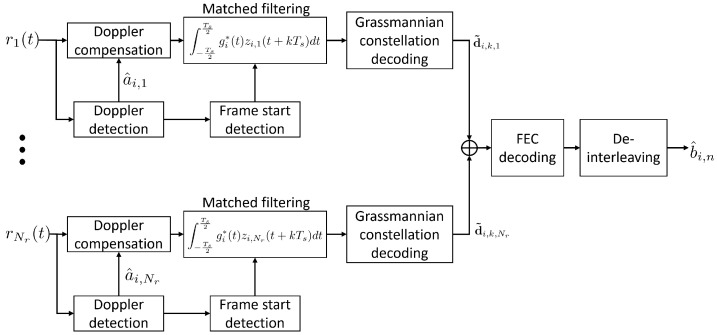
Receiver diagram.

A global diagram of the receiver is shown in Figure 3. The acoustic signals sent by the $N_{u}$ users, received by an array of $N_{r}$ hydrophone sensors such as the signal recorded in baseband at the *p*-th sensor, can be expressed as [21]

(6) $$r_{p}\left(t\right)=\sum_{i=1}^{N_{u}}\int_{-\infty }^{+\infty }h_{i,p}\left(\tau ,t\right)s_{i}\left(\left(1-a_{i,p}\right)\left(t-\tau \right)\right)e^{j2\pi f_{c}a_{i,p}\left(t-\tau \right)}d\tau +n_{p}\left(t\right),$$

where $h_{i,p}\left(\tau ,t\right)$ is the time-varying channel impulse response between the *i*-th user and the *p*-th receiver, $f_{c}$ denotes the center frequency of the passband transmitted signal, $a_{i,p}=\frac{v_{i,p}}{c}$ is the motion-induced Doppler scale factor for the *i*-th user, with $v_{i}$ as the relative speed of the *i*-th user with respect to the receiver *p*, and c≃1500 m/s as the speed of sound in water. Finally, $n_{p}\left(t\right)$ is the assumed additive white Gaussian noise for the *p*-th receiver. If the Doppler shift can be estimated, the Doppler effect is usually removed prior to decoding by resampling the received baseband signal and compensating the phase rotation as follows [22]:

(7) $$z_{i,p}\left(t\right)=r_{p}\left(\frac{t}{1-{\hat{a}}_{i,p}}\right)e^{-j2\pi f_{c}\left(\frac{{\hat{a}}_{i,p}}{1-{\hat{a}}_{i,p}}\right)t},$$

where ${\hat{a}}_{i,p}$ denotes an estimation of the Doppler shift. By assuming perfect time symbol synchronization, the *k*-th information data $k\in [1,N_{s}]$ of the *i*-th user for the *p*-th receiver can be estimated by matched filtering $z_{i,p}\left(t\right)$ with the transmit waveform of user *i* as follows:

(8) $$\begin{array}{cc} {\tilde{d}}_{i,k,p} & =\max_{k\frac{T_{s}}{2}\leq t\leq \left(k+1\right)\frac{T_{s}}{2}}\left[\int_{-\infty }^{+\infty }g_{i}^{*}\left(-u\right)z_{i,p}\left(t-u\right)du\right] \end{array}$$

(9) $$\begin{array}{cc} \; & =\int_{-\frac{T_{s}}{2}}^{\frac{T_{s}}{2}}g_{i}^{*}\left(t\right)z_{i,p}\left(t+kT_{s}\right)dt \end{array}$$

(10) $$\begin{array}{cc} \; & =\gamma_{i,k,p}d_{i,k,p}+\eta_{i,k,p}+w_{i,k,p}, \end{array}$$

where $\gamma_{i,k,p}$ denotes the bias of the decoder, $\eta_{i,k,p}$ is the interference terms, and $w_{i,k,p}$ is the additive noise term for the *p*-th receiver; the exact expression of these three terms is provided in Appendix A. The final symbol estimation and decision is obtained by combining the $N_{r}$ received signals as follows:

(11) $${\tilde{d}}_{i,k}=\sum_{p=1}^{N_{r}}{\tilde{d}}_{i,k,p}.$$

As explained in [23], if the modulation duration $T_{s}$ is greater than the UWA channel delay spread, the inter-symbol interference is negligible. On the other hand, due to the mutual orthogonality property of $g_{i}\left(t\right)$, the multi-user interference is also limited to obtain $\eta_{i,k,p}$ terms that decay towards 0 as $T_{s}$ tends to +∞.

In [12], data symbols $d_{i,k}$ belong to a DPSK modulation constellation in order to avoid the use of channel-estimation-based equalizers at the receiver side to compensate for the channel coefficients $\gamma_{i,k,p}$. A UWA communication channel may have rapid time variations, and differential modulation schemes have been demonstrated to provide good reliability vs. spectral efficiency trade-offs and, in some conditions, even outperform coherent modulation [24]. However, the main drawback of a DPSK modulation lies in its lower robustness against additive noise with respect to a Phase Shift Keying (PSK) modulation that limits its spectral efficiency at the same Bit Error Rate (BER) [25]. In the following, we will consider an alternative to the DPSK constellation by considering a Grassmannian constellation.

## 3. Grassmannian Constellations

In the literature, many Grassmannian constellation designs have been proposed to define the different $d_{i,k}$ [26]. Each Grassmannian constellation has its own method of symbol generation and decoding. In general, a good constellation should consist of codewords maximally distant from each other, while also ensuring sphere-packing properties, which translates in evenly spread symbols over a Grassmannian manifold. For example, in [27], the distance between codewords is maximized by a numerical optimization. This optimization generates a lack of structure in the modulation that requires that the codeword be stored on reception. To avoid this storage, codewords should be generated to have a given structure. For example, in [13,14,26], a new Grassmannian-structured constellation is generated through Cube-Split algorithms, whereby the Grassmannian space is partitioned into cells.

The main advantage of this new constellation lies in its robustness against single tap channel fading that causes such modulation to be decoded without Channel State Information (CSI) at the receiver and as a consequence without a complex equalization stage as in, for example, Quadrature Amplitude Modulation (QAM). Moreover, the advantage of Grassmannian constellation with respect to conventional Differential PSK modulation lies in its higher spectral efficiency that is achieved by a multi-level constellation format. To begin with, the encoded bit stream will be split into two parts. One part represents the number of the different cells of the Grassmannian space, while the other part will provide a set of coordinates corresponding to each cell of the Grassmannian space. The final constellation symbol is given in the form of a vector with *M* components.

In the following, we will detail the Cube-Split modulation design and the decoding process for a spread-spectrum communication scheme. In [26], the communication channel is a flat Rayleigh fading channel. In our study, we consider the underwater acoustic channel that is considered to be a non-flat fading channel [28], and the different users will be moving at a speed unknown by the receiver.

### 3.1. Transmitter Modulation Scheme Design

Let $G(C^{M},1)$ be the Grassmannian manifold with $M=2^{n}$ and $n\in N^{*}$. A Grassmannian constellation symbol will be defined by a vector $d_{i,k}={\left[d_{i,k,1},d_{i,k,2},\ldots ,d_{i,k,M}\right]}^{T}\in C^{M\times 1}$ to remain consistent with Equation (1), where $i\in [1,N_{u}]$ is the user number and $k\in [1,N_{s}]$ is the processed constellation symbol. Each user will have the same modulation scheme and constellation. The general idea of the Cube-Split constellation is to partition the $G(C^{M},1)$ space into *M* cells and to define, in each of these cells, a local coordinate system using a bijective mapping. A Grassmannian constellation symbol is then defined by $N=\log_{2}\left(M\right)+2\left(M-1\right)L_{0}$ bits, where $\log_{2}\left(M\right)$ bits indicate the cell index, and $L_{0}\in N^{*}$ represents the number of local coordinates. The spectral efficiency of the Cube-split modulation is given by

(12) $$\eta =\frac{\log_{2}\left(M\right)+2\left(M-1\right)L_{0}}{M}.$$

In the following, we give an overview of the Cube-Split modulation. For more details, the reader can refer to [13,26]. The cell l∈[1,M] of $G(C^{M},1)$ is defined by

(13) $$C_{l}=\left\{x=\left(x_{1},\ldots ,x_{M}\right)\in G\left(C^{M},1\right):d\left(x,\xi_{l}\right)<d\left(x,\xi_{j}\right),\forall j\in \left[1,M\right]\setminus \left\{l\right\}\right\},$$

where

(14) $$d\left(x,\xi_{l}\right)=\sqrt{1-|{\sum_{q=1}^{M}x_{q}\xi_{l,q}|}^{2}}$$

is the chordal distance between x and $\xi_{l}$.

To simplify Equation (13), we define a center for each cell using the canonical basis vectors. Let $\left\{e_{1},\ldots ,e_{M}\right\}$ be the centers of the cells with $e_{l}$ as the M×1 canonical basis vector, with 1 at position *l* and 0 elsewhere. With this choice of vectors for the centers of the cells, Relation (13) can be simplified as [26]

(15) $$C_{l}=\{x=\left(x_{1},\ldots ,x_{M}\right)\in G\left(C^{M},1\right):\;|x_{l}\left|>\right|x_{j}|,\forall j\in \left[1,M\right]\backslash \left\{l\right\}\}.$$

The codewords of the constellation are mapped in the different cells $C_{l}$ with a bijective mapping whose definition is given in Appendix B. $\forall i\in [1,N_{u}]$, $\forall k\in [1,N_{s}]$:

(16) $$\phi_{l}:q_{i,k}\in \overset{2(M-1)}{\underset{j=1}{⨂}}A_{j}⟼\phi_{l}\left(q_{i,k}\right)\in C_{l},$$

where ⨂ is the Cartesian product, and $A_{j}$ is a subset of the interval [0,1] defined ∀j∈[1,2(M−1)] by [26]:

(17) $$A_{j}=\left\{\frac{1}{2^{L_{0}+1}},\frac{3}{2^{L_{0}+1}},\ldots ,\frac{2^{L_{0}+1}-1}{2^{L_{0}+1}}\right\},$$

with $L_{0}\in N^{*}$ representing the number of local coordinates.

The constellation can then be defined $\forall i\in [1,N_{u}]$, $\forall k\in [1,N_{s}]$ as

(18) $$D^{\left(i,k\right)}=\left\{d_{i,k}=\phi_{l}\left(q_{i,k}\right)|l\in \left[1,M\right],q_{i,k}\in \overset{2(M-1)}{\underset{j=1}{⨂}}A_{j},d_{i,k}\in C^{M\times 1}\right\}.$$

In spread-spectrum communication, for $i\in [1,N_{u}]$, $k\in [1,N_{s}]$ each element of the vector $d_{i,k}={\left[d_{i,k,1},\ldots ,d_{i,k,M}\right]}^{T}\in C^{M\times 1}$ will be spread by the waveform $g_{i}\left(t\right)$. To keep consistency with Equation (1), we define $\forall i\in [1,N_{u}]$:

(19) $$d_{i}={\left[d_{i,1},\ldots ,d_{i,N_{s}}\right]}^{T}={\left[\left[d_{i,1,1},\ldots ,d_{i,1,M}\right],\ldots ,\left[d_{i,N_{s},1},\ldots ,d_{i,N_{s},M}\right]\right]}^{T},$$

with $d_{i}\in C^{N_{s}M\times 1}$ and $\forall q\in [1,N_{s}M]$, $d_{i,q}=d_{i,k,l}$.

The expression of the signal transmitted in baseband for the Cube-Split modulation is then given by

(20) $$s_{i}\left(t\right)=\sum_{q=1}^{N_{s}M}d_{i,q}g_{i}\left(t-qT_{s}\right).$$

We can see that the difference comes from the upper bound of the sum, which is, in the case of the Cube-Split modulation, equal to $N_{s}M$.

### 3.2. Receiver Demodulation Scheme Design

For the demodulation process, Equation (8) represents the output after matched filter. The receiver knows the parameters *M* and $L_{0}$, which represent the modulation order and the number of local coordinates of the Cube-Split modulation. We can then define for the *p*-th receiver and $\forall i\in [1,N_{u}]$

(21) $${\tilde{d}}_{i,p}={\left[{\tilde{d}}_{i,1,p},\ldots ,{\tilde{d}}_{i,N_{s},p}\right]}^{T}={\left[\left[{\tilde{d}}_{i,1,1,p},\ldots ,{\tilde{d}}_{i,1,M,p}\right],\ldots ,\left[{\tilde{d}}_{i,N_{s},1,p},\ldots ,{\tilde{d}}_{i,N_{s},M,p}\right]\right]}^{T},$$

to remain consistent with Equation (19) and ${\tilde{d}}_{i,p}\in C^{N_{s}M\times 1}$. To find the symbol transmitted ${\tilde{d}}_{i,k,p}$ with $k\in [1,N_{s}]$, we proceed in two steps: we decode first the cell number and then the local coordinates.

We begin by calculating the left singular vector $u_{i,k,p}={\left[u_{i,k,1,p},\ldots ,u_{i,k,M,p}\right]}^{T}$ corresponding to the largest singular value of the received signal ${\tilde{d}}_{i,k,p}$ on the *p*-th hydrophone, which is also the solution for $k\in [1,N_{s}]$ of

(22) $$\arg\max_{u_{i,k,p}\in C^{M}:||u_{i,k,p}{\left|\right|}^{2}=1}|\sum_{l=1}^{M}{\tilde{d}}_{i,k,l,p}u_{i,k,l,p}{|}^{2}.$$

The cell index estimate is given by Equations (13) and (14):

(23) $${\hat{l}}_{i,k,p}=\arg\min_{l\in [1,M]}d\left(u_{i,k,p},e_{l}\right)=\arg\max_{l\in [1,M]}\left|u_{i,k,l,p}\right|.$$

The local coordinates are estimated by first applying the inverse mapping $\phi_{\hat{l}}^{-1}$ to have ${\tilde{q}}_{i,k,p}=\left[{\tilde{q}}_{i,k,1,p},\ldots ,{\tilde{q}}_{i,k,2M-2,p}\right]=\phi_{\hat{l}}^{-1}\left(u_{i,k,p}\right)$. The closest point to ${\tilde{q}}_{i,k,p}$ in ${⨂}_{j=1}^{2(M-1)}A_{j}$ is obtained for $\forall j\in A_{j}$ by

(24) $${\hat{q}}_{i,k,p}^{\left(j\right)}=\arg\min_{q\in A_{j}}\left|{\tilde{q}}_{i,k,p}^{\left(j\right)}-q\right|.$$

The decoded symbol ${\tilde{d}}_{i,k,p}$ is then identified from the estimated parameters $\left\{{\hat{l}}_{i,k,p},{\hat{q}}_{i,k,p}\right\}$ as ${\tilde{d}}_{i,k,p}=\phi_{{\hat{l}}_{i,k,p}}^{-1}\left({\hat{q}}_{i,k,p}\right)$. The final estimate symbol is obtained by combining the $N_{r}$ receive streams. $\forall i\in [1,N_{u}]$, $\forall k\in [1,N_{s}]$, we have

(25) $${\tilde{d}}_{i,k}=\sum_{p=1}^{N_{r}}{\tilde{d}}_{i,k,p},$$

with ${\tilde{d}}_{i,k}\in C^{M\times 1}$.

## 4. Results and Discussion

**Figure 4:**
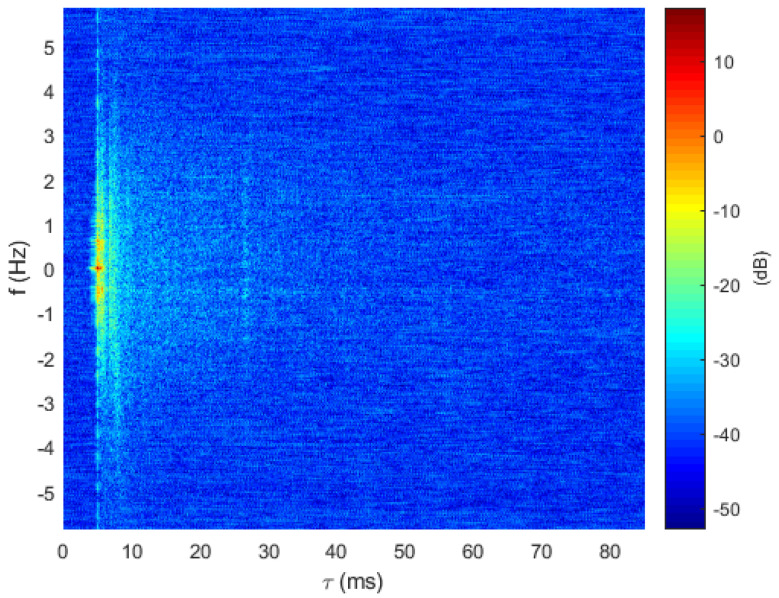
Delay-Doppler spread function extracted from channel sounding for $D_{1}=200$ m, roadstead of Brest, France.

The channel sounding experiments took place during summer 2019 in the roadstead of Brest, France, by using the IROMI-LMAIR platform [29]. At the transmitter side, one transducer immersed at a 2 m depth was used. At the receiver side, 5 hydrophone sensors were vertically deployed with a spacing of 1 m. Five channel soundings were performed at different transmission ranges $D_{i}$ between 65 and 540 m by using a 511-Maximal Length Sequence (MLS) as a probe signal [30] centered on $f_{c}=27$ kHz over a 4 kHz bandwidth. The different channel soundings are not calibrated with respect to propagation loss as a near-far effect. Time synchronization among the hydrophone channels is preserved. Figure 4 provides an example of the delay-Doppler spread extracted from the successive estimated CIR. Estimated channel delay and Doppler spreads are reported in Table 1.

### 4.1. Watermark Replay Channel

To emulate a realistic transmission experiment, we take as a basis the Watermark simulator [17] that is a replay channel simulator driven by measurements of time varying CIR. The principle of the simulator consists in distorting input waveforms by convolving them with measured channels. To simulate a realistic mobile multiuser communication, we considered the following procedure: (i) replay sounded channels on the pass-band signal transmitted by each user $s_{i}\left(t\right)$ by using the Watermark simulator, (ii) simulate the Doppler effect linked on the relative motion of each user, (iii) delay and scale the signal according to the relative range of each user in order to represent attenuation and delays of each user signal, and (iv) sum all signals obtained in order to represent a multi-user transmission. As a result, the final signal obtained on the *p*-th hydrophone can be expressed in baseband as

(26) $$\begin{array}{cc} r_{p}\left(t\right) & =\sum_{i=1}^{N_{u}}\gamma_{i}\int_{-\infty }^{+\infty }{\hat{h}}_{i,p}\left(\tau ,t\right)s_{i}\left(\left(1-a_{i,p}\right)\left(t-\tau -{\bar{\tau }}_{i}\right)\right)e^{j2\pi f_{c}a_{i,p}\left(t-\tau \right)}d\tau +n_{p}\left(t\right), \end{array}$$

where the Doppler shift is artificially added in the receive signal by resampling and phase rotating the transmitted signal $s_{i}\left(t\right)$, ${\hat{h}}_{i,p}\left(\tau ,t\right)$ denotes the recorded CIR of the *i*-th user to the *p*-th hydrophone, ${\bar{\tau }}_{i}$ is the communication delay between the *i*-th user and the receiver, and $n_{p}\left(t\right)$ is the additive noise on the *p*-th hydrophone assumed to be white, zero mean, and Gaussian distributed. $\gamma_{i}$ represents the propagation losses that generate the near-far effect in the case of multi-user communication. The value is assumed to be the same for each receiving hydrophone and is given by [2]

(27) $$\gamma_{i}=\sqrt{10^{-A(D_{i},f_{c})/10}},$$

where $A(D_{i},f_{c})$ denotes the acoustic power attenuation in decibels at range $D_{i}$ and frequency $f_{c}$:

(28) $$A\left(D_{i},f_{c}\right)=10\times m\log_{10}\left(D_{i}\right)+\alpha \left(f_{c}\right)\cdot 10^{-3}\cdot D_{i},$$

with *m* as the spatial dispersion factor that is assumed to be cylindrical (m=1), and $\alpha (f_{c})$ is given by Thorp’s formula [31]. The Signal-to-Noise Ratio (SNR) in dB is then defined $\forall p\in [1,N_{r}]$:

(29) $${\mathrm{SNR}}_{p}=10\log_{10}\left\{\frac{E\left\{|\sum_{i=1}^{N_{u}}\gamma_{i}\int_{-\infty }^{+\infty }{\hat{h}}_{i,p}\left(\tau ,t\right)s_{i}\left(\left(1-a_{i}\right)\left(t-\tau -{\bar{\tau }}_{i}\right)\right)e^{j2\pi f_{c}a_{i}\left(t-\tau \right)}d\tau {|}^{2}\right\}}{E\left\{|n_{p}\left(t\right){|}^{2}\right\}}\right\}.$$

In the following, we will take into account the problems of frame synchronization and Doppler shift estimation at the receive side. The estimation of both the user frame starting time and the Doppler shift is performed owing to a preamble known by the receiver. More especially, Doppler estimation is obtained with a Doppler-bank matched filter, cycling through velocities from [−2,2] m/s in steps of 0.1 m/s [10]. The filter is matched to the signal preambles that are chosen as a PN sequence or MU-HFM waveform. Below, we recall the expression of PN sequence-based waveforms:

(30) $$g_{{pr}_{i}}\left(t\right)=\sum_{l=0}^{N_{SF_{pr}}-1}c_{i,l}\phi \left(t-lT_{c}\right),$$

with $\left[c_{i,1},c_{i,2},\ldots ,c_{i,N_{SF_{pr}}}\right]$ as the spreading code of length $N_{SF_{pr}}$, where $T_{c}$ is the chip duration, $N_{SF_{pr}}$ is the spreading factor, and ϕ(t) is the pulse shaping filter chosen as a Square Root Raised Cosine (SRRC) filter [25].

Transmission system parameters are provided in Table 2, where parameter ζ of MU-HFM is computed according to the signal bandwidth by using Simpson’s method. For B=4 kHz, we choose [23]

(31) $$\zeta =\left\{\begin{array}{cc} 0.0214 & \mathrm{if}\;i\;\mathrm{is}\;\mathrm{even}\\ -0.0214 & \mathrm{otherwise}. \end{array}\right.$$

### 4.2. Performance Metrics

In order to measure the efficiency of the proposed transmission scheme, we choose as a performance metric the average effective data rate defined as follows [23,25]:

(32) $$D_{e}^{\mathrm{MU}-\mathrm{HFM}}=\frac{R_{C}\eta }{\left(T_{s}+T_{g_{pr}}+T_{pr}\right)}\cdot \left(1-\mathrm{FER}\right)\;\left[\mathrm{bps}\right],$$

where η denotes the spectral efficiency of the considered modulation defined as

(33) $$\eta =\left\{\begin{array}{cc} 2 & \mathrm{DQPSK}\;\mathrm{modulation}\\ \frac{\log_{2}\left(M\right)+2\left(M-1\right)L_{0}}{M} & \mathrm{Grassmannian}\;\mathrm{modulation}, \end{array}\right.$$

where $R_{C}$ denotes the channel coding rate, and FER is the Frame Error Rate. In the following, a frame is considered erroneous when at least one bit per frame after channel decoding is erroneous.

As a benchmark protocol, we consider a conventional deterministic multiple access scheme for the UWA channel, i.e., TDMA and CDMA. In order to provide a fair comparison, the TDMA protocol is combined with Direct Sequence Spread Spectrum (DSSS) modulation with a spreading factor $N_{SF}$ equal to the CDMA one and equal to the time-bandwidth product of MU-HFM waveforms, such as the performance of all protocols that are comparable in the single user scenario. In order to prevent intersymbol interference, the symbol duration $T_{s}$ and the TDMA guard interval time $T_{g}$ are both chosen to be greater than the maximum duration of the various channel delays reported in Table 1. For each protocol, we consider the conventional Differential Quaternary Phase Shift Keying (DQPSK) constellation and the proposed Grassmannian modulation. The average effective data rate per user for CDMA and TDMA is given by

(34) $$D_{e}^{\mathrm{CDMA}}=\frac{R_{C}\eta }{\left(N_{SF}\cdot T_{c}+T_{g_{pr}}+T_{pr}\right)}\cdot \left(1-\mathrm{FER}\right)\;[\mathrm{bps}],$$

(35) $$D_{e}^{\mathrm{TDMA}}=\frac{R_{C}\eta }{N_{u}\left(N_{SF}T_{c}+T_{g_{pr}}+T_{pr}\right)+\left(N_{u}-1\right)T_{g}}\cdot \left(1-\mathrm{FER}\right)\;[\mathrm{bps}].$$

Finally, to evaluate the accuracy of the Doppler shift estimation ${\hat{a}}_{i,p}$, we use the Root Mean Square Error (RMSE) metric defined $\forall i\in [1,N_{u}]$, $\forall p\in [1,N_{r}]$ as

(36) $${\mathrm{RMSE}}_{i,p}=\sqrt{E(|{\hat{a}}_{i,p}-a_{i,p}{|}^{2})}.$$

### 4.3. Performance Results

Compared to [12], in this part, we provide the performance results of the 3 studied protocols with and without Grassmannian modulation by using the UWA replay channel described before and by considering mobile AUVs, whose motion is emulated by adding the motion-induced Doppler scale at the output of the Watermark channel. For each frame, the speed value of each AUV is randomly selected in the interval [−2,2] m/s and assumed constant along a frame. Moreover, for all simulations, frame synchronization and Doppler shift estimation are performed at the receive side in order to be as close as possible to a real experiment.

**Figure 5:**
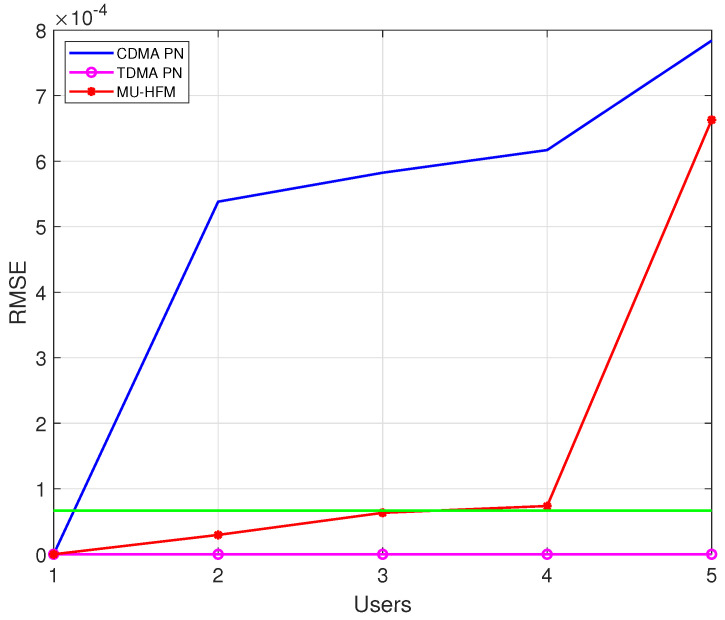
RMSE for Doppler shift estimation based on the number of users for different types of preamble of a 63.75 ms duration for 5000 frames.

Figure 5 shows the RMSE of the Doppler shift estimation as a function of the number of users. As a reference, we plot, in a green line, an estimation error of 0.1 m/s, leading to a Doppler shift error of $0.6\times 10^{-4}$, which represents the search step of the Doppler bank algorithm. We can see for a simultaneous communication that a preamble based on a PN sequence is not efficient since, from 2 users, the estimation is strongly degraded. This is due to the low resistance of the PN sequence against channel impairments. However, by using a MU-HFM-based preamble, the accuracy is satisfactory up to 4 users. At 5 users, the Doppler estimation is substantially erroneous, yielding a strong degradation of the decoding performance. As a reference, we can see that the TDMA-PN curve representing a PN preamble-based Doppler estimation in the TDMA protocol case leads to perfect Doppler estimation, regardless of the number of users.

**Figure 6:**
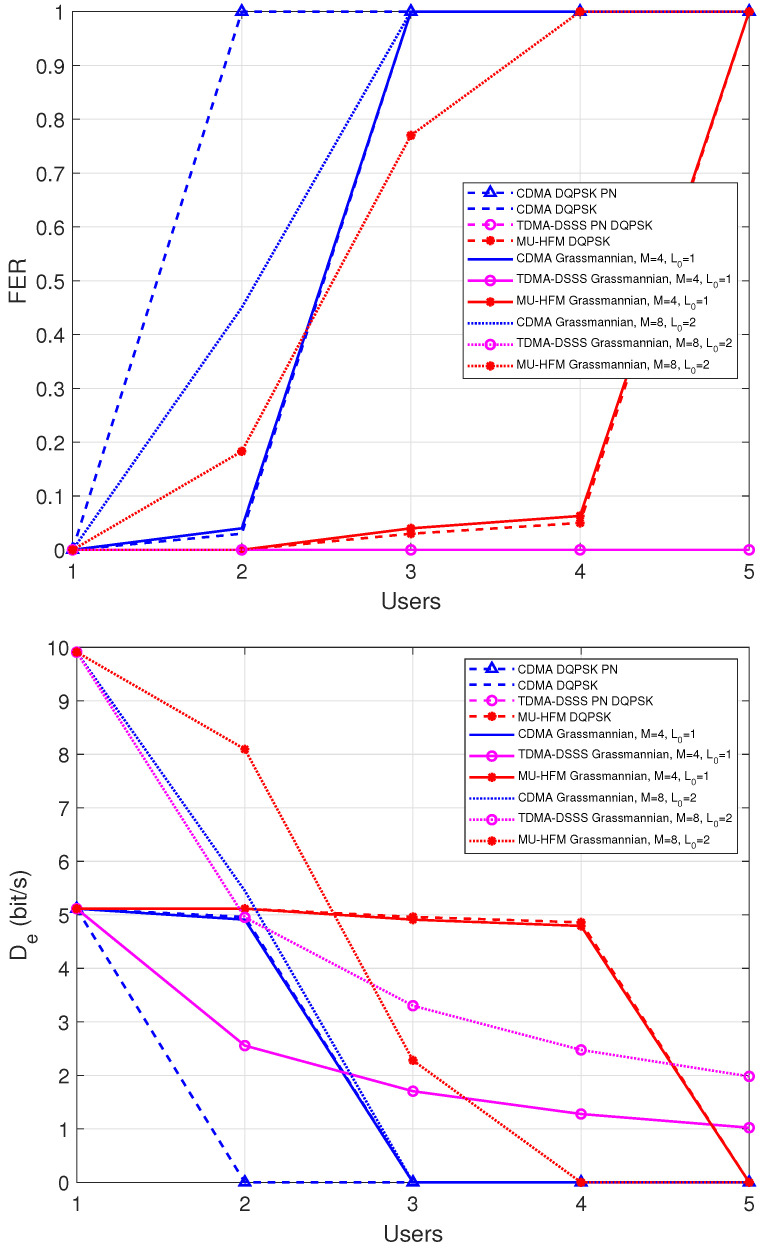
AverageFrame Error Rate (FER) performance (**up**) and effective data rate per user (**down**) versus the number of users for the replayed channel of the roadstead of Brest, where $N_{r}=1$ sensor, and average SNR = 10 dB. The abbreviation PN means that a PN code type preamble was used; in the other cases, a MU-HFM type preamble was considered.

In Figure 6, FER and effective data rates results are presented by assuming a single stream receiver. For each protocol, we can see that DQPSK and Grassmannian modulation with M=4 provide the same FER. Logically, by increasing the parameter *M* of the Grassmannian modulation to M=8, the effective data rate is increased, since the number of local coordinates is also higher. However, the Grassmannian modulation requires precise decoding based on the index of the cell and on the local coordinates associated with the cell, so the increase in *M* makes the modulation less robust to noise and interference leading to higher FER. The CDMA-PN curve that represents the performance of CDMA with a PN sequence as a preamble are provided in order to show the ineffectiveness of this type of preamble in the case of simultaneous communication. As shown in Figure 5, this is due to the very low accuracy in the estimation of the Doppler shift. By comparing protocols associated with MU-HFM preambles, we can see that the maximum number of simultaneous users is 4. Beyond that, the Doppler shift estimate is too erroneous, as shown in Figure 5. The CDMA approach appears less efficient beyond 3 active users, whereas the traditional TDMA provides the best performance at 5 users. This poor performance of CDMA compared to that of MU-HFM is explained by the fact that, in MU-HFM, we have achieved an orthogonal combination using the Gram–Schmidt method between an HFM signal and a set of narrowband chirps. From 4 users, the MU-HFM has an effective data rate that is decreasing due to the higher multiuser interference terms yielding a higher FER. For the Grassmannian modulation with M=8, we can see the MU-HFM still has the best performance compared to CDMA for the same reasons as found previously.

**Figure 7:**
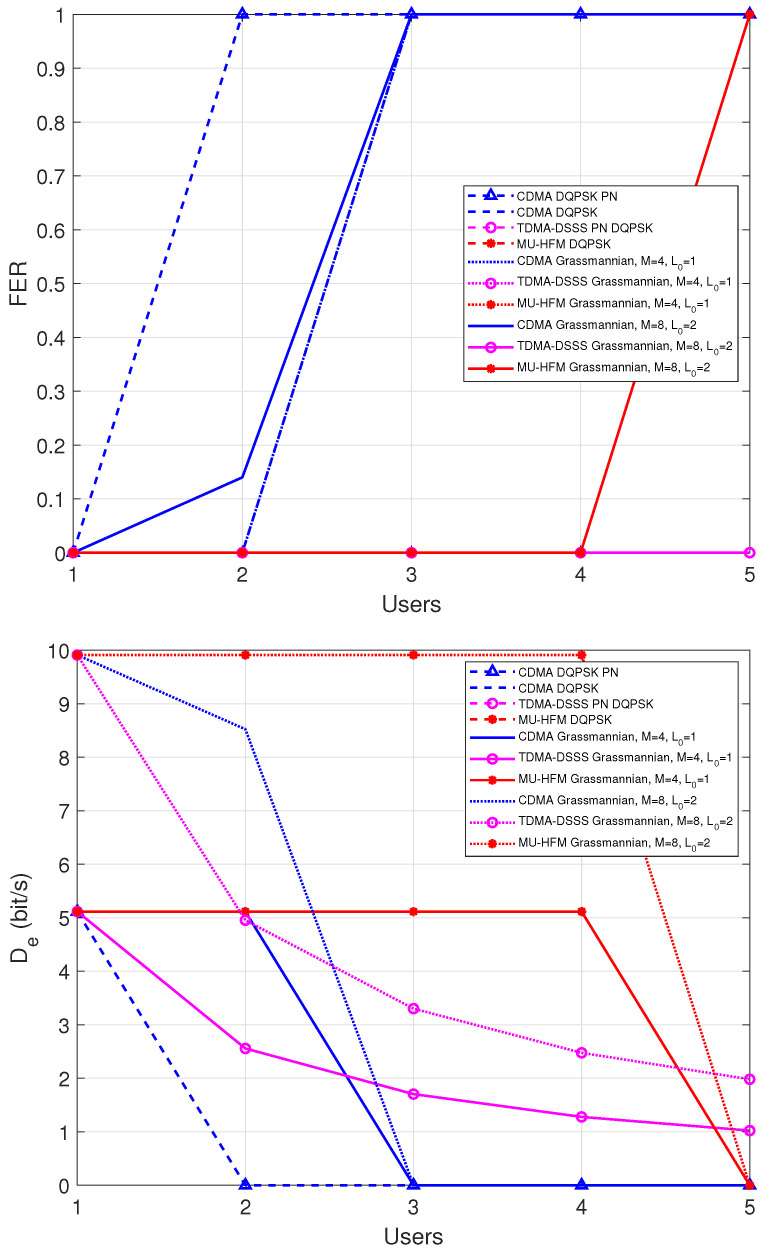
AverageFER performance (**up**) and effective data rate per user (**down**) versus the number of users for the replayed channel of the roadstead of Brest, where $N_{r}=5$ sensors, and average SNR = 10 dB. The abbreviation PN means that a PN code type preamble was used; in the other cases, a MU-HFM type preamble was considered.

In Figure 7, results are presented with 5 hydrophones at the receiver side and multi-channel processing, as shown in (11). As for the system with $N_{r}=1$, the maximum number of users is 4 for protocols with a MU-HFM type preamble. For a preamble with a PN sequence, CDMA is only possible for 1 user. However, the preamble with a PN sequence allows TDMA communication up to 5 users because the estimation of the Doppler shift is perfect. In the case of the CDMA protocol with an MU-HFM preamble, the FER of the Grassmannian modulation with M=8 is slightly improved for 2 users. In the case of MU-HFM protocol, the FER performance of both differential and Grassmannian modulations are substantially improved, yielding error free transmission up to 4 users with DQPSK or Grassmannian modulation with M=4. With M=8, error free transmission is reached for up to 4 users and again at 5 users. The transmission is ineffective due to the poor estimation of the Doppler shift. By comparison with Figure 6, this demonstrates that the MU-HFM protocol takes full advantage of the spatial processing gain provided by the multi-sensor architecture. On the other hand, the MU-HFM combined with Grassmannian modulation with M=8 offers a constant user data rate of 10 bps for up to 4 mobile users, which demonstrates the superiority of the proposed MU-HFM protocol in comparison to conventional protocols. These performance results also show the spectral efficiency gain of Grassmannian modulation against differential modulation. In fact, in order to reach a similar data rate, the MU-HFM should be combined with a 16-state DPSK modulation that is clearly not feasible in the context of UWA communication.

## 5. Conclusions

In this paper, we have considered the use of a Grassmannian modulation, named Cube-Split modulation, as an alternative to differential modulation for multiuser communication in a scenario where an AUV fleet transmits acoustic data to a surface receiver within the same bandwidth. To reduce the detrimental effects of the UWA channel, we have considered spread-spectrum-based communications—more specifically, MU-HFM waveforms designed for multiple access in the UWA channel. Experimental results with the Watermark channel fed by sea channel sounding demonstrate that Grassmannian modulation outperforms conventional quaternary differential modulation when multi-hydrophone reception is considered. In particular, the MU-HFM offers a quasi-constant effective data rate as the number of active users increases, up to a maximum of 4 users. Beyond that, the estimation of the Doppler shift is no longer possible for a preamble composed of the MU-HFM waveform. Consequently, the use of Grassmannian modulation is demonstrated to be an efficient alternative of differential modulation that cannot exceed 8 states in practice. Indeed, to reach an equivalent data rate, coherent QAM modulation would be required, which implies an equalization step that eliminates the interference between symbols. However, such an approach would require precise estimates of multiuser channels based on a pilot sequence designed with respect to the channel coherence time that would limit the number of simultaneous users.

## Figures and Tables

**Table 1:** Watermark channel parameters.

Symbol	Signification	Value
$f_{c}$	Center frequency	27 kHz
$f_{s}$	Sampling frequency	96 kHz
*B*	Signal bandwidth	4 kHz
$D_{i}$	Transmission range	[65, 540] m
$z_{w}$	Water depth	10 m
SNR	Signal to noise ratio	10 dB
$\tau_{max}$	RMS channel delay spread [30]	[8.85, 26.49] ms
$\sigma_{max}$	RMS channel Doppler spread [30]	[0.85, 2.9] Hz

**Table 2:** System parameters.

Symbol	Signification	Value
*M*	Grassmannian modulation order	4, 8
$L_{0}$	Number of local coordinates	1, 2
$N_{r}$	Number of hydrophone receiving	5
$N_{s}$	Number of symbols per frame	200
$N_{f}$	Number of frames	5000
C	FEC code type	Convolutive code
$g_{C}$	FEC code generator	(133, 171)_*o*_
$R_{C}$	FEC code rate	$\frac{1}{2}$
$T_{g}$	Guard interval time TDMA	31.3 ms
$T_{c}$	Chip duration	0.25 ms
$f_{l}$, $f_{h}$	Bounds of HFM signal	6 kHz, 10 kHz
α	Pulse shaping filter roll-off factor	0.25
$T_{s}$	Symbol duration	31.75 ms
$N_{SF}$	Spreading factor	127
$T_{pr}$	Preamble duration	63.75 ms
$N_{SF_{pr}}$	Spreading factor for the preamble	255
$T_{g_{pr}}$	Guard interval time between the preamble and the message	100 ms

## Abbreviations

The following abbreviations are used in this manuscript:

AUV	Autonomous Underwater Vehicle
AWGN	Additive White Gaussian Noise
BER	Bit Error Rate
CP	Cyclic Prefix
CDMA	Code-Division Multiple Access
CIR	Channel Impulse Response
CSI	Channel State Information
CSMA	Carrier Sense Multiple Access
PSK	Phase Shift Keying
DFE	Decision Feedback Equalizer
DPSK	Differential Phase Shift Keying
DBPSK	Differential Binary Phase Shift Keying
DQPSK	Differential Quaternary Phase Shift Keying
DSSS	Direct Sequence Spread Spectrum
FDMA	Frequency Divsion Multiple Access
FEC	Forward Error Correction
FER	Frame Error Rate
FFT	Fast Fourier Transform
FSK	Frequency Shift Keying
HFM	Hyperbolically Frequency Modulation
ISI	Inter Symbol Interference
MC-CDMA	Multi-Carrier Code-Division Multiple Access
MLS	Maximal Length Sequence
CSS	Chirp Spread Spectrum
LFM	Linear Frequency Modulation
MU-CSS	MultiUser Chirp Spread Spectrum
MU-HFM	MultiUser Hyperbolically Frequency Modulation
MU-MIMO	multiuser Multiple-Input Multiple-Output
OFDM	Orthogonal Frequency Division Multiplex
PN	Pseudo-Noise
PPC	Passive Phase Conjugation
QAM	Quadrature Amplitude Modulation
R_c	FEC rate
RMS	Root Mean Square
SIMO	Single Input Multiple Output
SISO	Single Input Single Output
SINR	Signal-to-Interference-plus-Noise Ratio
SNR	Signal-to-Noise Ratio
SRRC	Square Root Raised Cosine
TDMA	Time-Division Multiple Access
UAC	Underwater Acoustic channel
UWA	Underwater Acoustic
Watermark	underWater AcousTic channEl Replay benchMARK
VTRM	Virtual Time Reversal Mirror
FrFT	Fractional Fourier Transform
RMSE	Root Mean Square Error

## Appendix A. Calculation of *γ_i,k,p_*, *η_i,k,p_* and *w_i,k,p_*

In Section 2, the received baseband signal after Doppler pre-processing at the *p*-th receiver can be expressed as

(A1) $$\begin{array}{cc} z_{i,p}\left(t\right) & =r_{p}\left(\frac{t}{1-a_{i}}\right)e^{-j2\pi f_{c}\left(\frac{a_{i}}{1-a_{i}}\right)t} \end{array}$$

(A2) $$\begin{array}{cc} \; & =\left(\sum_{j=1}^{N_{u}}\int_{-\infty }^{+\infty }h_{j,p}\left(\tau ,\frac{t}{1-a_{i}}\right)s_{j}\left(\left(1-a_{j}\right)\left(\frac{t}{1-a_{i}}-\tau \right)\right)e^{j2\pi f_{c}a_{j}\left(\frac{t}{1-a_{i}}-\tau \right)}d\tau \right) \end{array}$$

(A3) $$\begin{array}{cc} \; & \;e^{-j2\pi f_{c}\left(\frac{a_{i}}{1-a_{i}}\right)t}+n_{p}\left(\frac{t}{1-a_{i}}\right)e^{-j2\pi f_{c}\left(\frac{a_{i}}{1-a_{i}}\right)t}. \end{array}$$

The combination of (1) and (10) yields

(A4) $$\gamma_{i,k,p}=\int_{\frac{-T_{s}}{2}}^{\frac{T_{s}}{2}}\int_{-\infty }^{+\infty }h_{i,p}\left(\tau ,\frac{t+kT_{s}}{1-a_{i}}\right)g_{i}^{*}\left(t\right)g_{i}\left(t-(1-a_{i})\tau \right)e^{-j2\pi f_{c}a_{i}\tau }d\tau dt.$$

(A5) $$\begin{array}{cc} \eta_{i,k,p} & =\sum_{\begin{array}{c} n=1\\ n\neq k \end{array}}^{N_{s}}d_{i,n}\int_{\frac{-T_{s}}{2}}^{\frac{T_{s}}{2}}\int_{-\infty }^{+\infty }h_{i,p}\left(\tau ,\frac{t+kT_{s}}{1-a_{i}}\right)g_{i}^{*}\left(t\right)g_{i}\left(t-\tau -\left(n-k\right)T_{s}\right)e^{-j2\pi f_{c}a_{i}\tau }d\tau dt \end{array}$$

(A6) $$\begin{array}{cc} \; & +\sum_{\begin{array}{c} j=1\\ j\neq i \end{array}}^{N_{u}}\sum_{n=1}^{N_{s}}d_{j,n}\int_{\frac{-T_{s}}{2}}^{\frac{T_{s}}{2}}\int_{-\infty }^{+\infty }h_{j}\left(\tau ,\frac{t+kT_{s}}{1-a_{i}}\right)g_{i}^{*}\left(t\right)g_{j}\left(\left(1-a_{j}\right)\left(\frac{t+kT_{s}}{1-a_{i}}-\tau \right)-nT_{s}\right) \end{array}$$

(A7) $$\begin{array}{cc} \; & \;e^{-j2\pi f_{c}\left(\frac{a_{i}-a_{j}}{1-a_{i}}\left(kT_{s}+t\right)+a_{j}\tau \right)}d\tau dt, \end{array}$$

and

(A8) $$w_{i,k,p}=e^{-j2\pi f_{c}\frac{a_{i}}{1-a_{i}}kT_{s}}\left(\int_{\frac{-T_{s}}{2}}^{\frac{T_{s}}{2}}g_{i}^{*}\left(t\right)n_{p}\left(\frac{t+kT_{s}}{1-a_{i}}\right)e^{-j2\pi f_{c}\left(\frac{a_{i}}{1-a_{i}}\right)t}dt\right).$$

## Appendix B. Definition of the Bijective Mapping ***ϕ***_l_ for the Cube-Split Modulation

To define the bijective mapping $\phi_{l}$ of Section 3, we will use an intermediate space that is given by the diagram below [26]:

(A9) $$\overset{2(M-1)}{\underset{j=1}{⨂}}A_{j}⟶D{\left(0,1\right)}^{M-1}⟶C_{l}.$$

where ⨂ is the Cartesian product, D(0,1)={z∈C:|z| < 1} is the unit disc, $A_{j}$ is defined by Equation (17), and $C_{l}$ is given by Equation (15).

With the scheme (A9), we equivalently define the bijective mapping $\phi_{l}$ through a mapping $\psi_{M-1}:q_{i,k}\in {⨂}_{j=1}^{2(M-1)}A_{j}⟼t_{i,k}={\left[t_{i,k,1},...,t_{i,k,M-1}\right]}^{T}\in D{\left(0,1\right)}^{M-1}$ with $i\in [1,N_{u}]$, $k\in [1,N_{s}]$. We recall that $N_{u}$ represents the number of users and that $N_{s}$ is the number of modulation symbols.

(A10) $$\phi_{l}\left(q_{i,k}\right)=\frac{1}{\sqrt{1+\sum_{l=1}^{M-1}{\left|t_{i,k,l}\right|}^{2}}}{\left[t_{i,k,1},...,t_{i,k,l-1},1,t_{i,k,l},\ldots ,t_{i,k,M-1}\right]}^{T}.$$

For the particular case M=2, we have $\forall i\in [1,N_{u}]$, $\forall k\in [1,N_{s}]$:

(A11) $$\psi_{1}\left(q_{i,k}\right)=\sqrt{\frac{1-e^{-\frac{|\omega_{i,k}{|}^{2}}{2}}}{1+e^{-\frac{|\omega_{i,k}{|}^{2}}{2}}}}\frac{\omega_{i,k}}{|\omega_{i,k}|},$$

where $\omega_{i,k}=N^{-1}\left(q_{i,k,1}\right)+jN^{-1}\left(q_{i,k,2}\right)$, with N(x) as the cumulative distribution function of the standard real univariate Gaussian given by

(A12) $$N\left(x\right)=\frac{1}{\sqrt{2\pi }}\int_{-\infty }^{x}e^{-\frac{\eta^{2}}{2}}d\eta ,$$

If M>2, we have $\forall i\in [1,N_{u}]$, $\forall k\in [1,N_{s}]$:

(A13) $$\psi_{M-1}={\left[\psi_{1}\left({\left[q_{i,k,1},q_{i,k,2}\right]}^{T}\right),\ldots ,\psi_{1}\left({\left[q_{i,k,2M-3},q_{i,k,2M-2}\right]}^{T}\right)\right]}^{T}.$$

The definition of $\psi_{M-1}$ allows us to define the inverse of $\phi_{l}$, which is to say, $q_{i,k}=\phi_{l}^{-1}\left(d_{i,k}\right)$, with $d_{i,k}={\left[d_{i,k,1},d_{i,k,2},\ldots ,d_{i,k,M}\right]}^{T}\in C^{M\times 1}$ as the Grassmannian modulation symbols. $\forall i,j,k\in \left[1,N_{u}\right]\times \left[0,M-1\right]\times \left[1,N_{s}\right]$:

(A14) $$\left\{\begin{array}{c} q_{i,k,2j-1}=N\left(ℜ\left(\omega_{i,k,j}\right)\right)\\ q_{i,k,2j}=N\left(ℑ\left(\omega_{i,k,j}\right)\right), \end{array}\right.$$

where

(A15) $$\omega_{i,k,j}=\sqrt{2\log\frac{1+|t_{i,k,j}{|}^{2}}{1-|t_{i,k,j}{|}^{2}}}\frac{t_{i,k,j}}{|t_{i,k,j}|},$$

(A16) $$t_{i,k}={\left[\frac{d_{i,k,1}}{d_{i,k,l}},\ldots ,\frac{d_{i,k,l-1}}{d_{i,k,l}},\frac{d_{i,k,l+1}}{d_{i,k,l}},...,\frac{d_{i,k,M}}{d_{i,k,l}}\right]}^{T}.$$

We then obtain Equation (18).
